# Novel mutations of maternal effect gene thyroid hormone receptor interactor 13 involved in biparental complete hydatidiform mole

**DOI:** 10.1097/MD.0000000000045419

**Published:** 2025-10-24

**Authors:** Qitao Zhan, Xinyun Yang, Yan Wang

**Affiliations:** a Department of Reproductive Endocrinology, Key Laboratory of Reproductive Genetics of National Ministry of Education, Women’s Hospital, Zhejiang University School of Medicine, Hangzhou, China; b Department of Pathology, Women’s Hospital, Zhejiang University School of Medicine, Hangzhou, China.

**Keywords:** BiCHM, hydatidiform mole, recurrent pregnancy loss, *TRIP13*, whole-exome sequencing

## Abstract

**Rationale:**

Biparental complete hydatidiform mole (BiCHM) is a rare form of molar pregnancy, frequently associated with familial recurrence, whose mechanism was historically unclear. It is classically diploid with biparental inheritance, but manifests as a complete hydatidiform mole (CHM). Cytogenetic studies suggested a link to maternal mutations in imprinted genes NOD-like receptor family, pyrin domain containing 7 or Kelch helper domain containing 3-like within the oocyte. This case reports a novel gene association.

**Patient concerns:**

A 43-year-old woman presented with a history of recurrent abnormal pregnancies: 2 CHM and 1 spontaneous abortion following an intracytoplasmic sperm injection (ICSI)-assisted conception.

**Diagnoses:**

Short tandem repeat polymorphism analysis of products of conception (including both CHM and the aborted ICSI pregnancy) revealed all 3 were biparental triploids derived from the patient and her husband. Whole-exome sequencing of the patient’s family identified that the patient carries compound heterozygous mutations (c.-82G > A [paternally inherited] and c.*106G > A [maternally inherited]) in the autosomal recessive gene thyroid hormone receptor interactor 13 (*TRIP13*), confirmed by Sanger sequencing.

**Interventions:**

Given the genetic findings and history of recurrent BiCHM, ovarian donation or adoption was recommended as the treatment strategy to achieve a normal pregnancy.

**Outcomes:**

The genetic diagnosis was established, informing future reproductive counseling and management options.

**Lessons:**

To our knowledge, this is the first report linking biallelic *TRIP13* mutations to recurrent BiCHM, indicating its potential role in the pathogenesis. ICSI-assisted reproduction did not improve pregnancy outcomes. Ovarian donation or adoption was recommended.

## 1. Introduction

Complete hydatidiform mole (CHM) is a disease that can occur during pregnancy, and the majority of these cases are androgenetic in nature, meaning that they originate from the father’s genetic material alone. However, there exists a less common form known as biparental CHM (BiCHM), which is classically diploid with biparental inheritance but manifests as a CHM.

At present, there are still limitations in diagnosing the types of hydatidiform moles based solely on ultrasound and pathological diagnosis. Short tandem repeat (STR) has its advantages in diagnosing the types and origins of hydatidiform moles.^[1,2]^ DNA genotyping of STR can differentiate between complete to partial hydatidiform moles. CHMs are usually diploid, whereas partial hydatidiform moles are triploid. Additionally, STR testing can identify the genetic origin and is recognized as the gold standard for confirming the type of hydatidiform mole, helping to determine whether it is of paternal, maternal, or biparental origin. It provides strong support for the accurate diagnosis and treatments for hydatidiform moles.

BiCHM is often linked to familial recurrent hydatidiform moles, a condition where the same individual experiences at least 2 occurrences of hydatidiform moles. Studies have shown that 50% to 80% patients diagnosed with recurrent hydatidiform moles carry biallelic pathogenic variants in either the NOD-like receptor family, pyrin domain containing 7 (*NLRP7*) or the Kelch helper domain containing 3-like (*KHDC3L*) genes.^[3–5]^ These mutations result in the failure to establish paternal imprints properly in the oocyte, leading to the formation of biparentally derived but epigenetically abnormal embryos.^[6]^ However, to date, there have been no reports of additional genes associated with BiCHM. In the case at hand, we discovered novel mutations of a maternal effect gene, which was associated with BiCHM.

## 2. Case presentation

### 2.1. Patient information and therapeutic intervention

The study was approved by the Research Ethics Committee of Women’s Hospital, Zhejiang University School of Medicine, Hangzhou, China (IRB-20200207-R). The informed patient consent was obtained in this study.

A 43-year-old female patient presented with a history of recurrent adverse pregnancies. In 2014, she underwent dilation and curettage for the first hydatidiform mole following a natural pregnancy. Preoperative ultrasound indicated an intrauterine mass measuring 3.6 cm × 4.3 cm × 2.5 cm with honeycomb-like heterogeneous echoes, and the serum human chorionic gonadotropin (HCG) level was 47,742 IU/L. Postoperatively, weekly HCG follow-up was conducted until it showed a continuous decline, with 3 consecutive negative HCG results by the third postoperative month. Subsequently, HCG was monitored every 1 to 2 months until the 1-year follow-up period concluded. In 2018, she had her second hydatidiform mole after another natural pregnancy. Preoperative ultrasound indicated an intrauterine mass measuring 5.2 × 4.5 × 3.8 cm, and the serum HCG level was 1,33,537 IU/L. On the day following uterine curettage, the HCG level was 34,922 IU/L. Weekly HCG follow-up was conducted. However, 4 weeks postoperatively, HCG rose to 3129 IU/L. Meanwhile, abdominal computed tomography (CT) revealed an intrauterine mass measuring 1.7 × 1.4 × 1.0 cm, and chest CT showed multiple pulmonary nodules. Cranial CT was negative. The patient was subsequently diagnosed with gestational trophoblastic neoplasia (stage III: 2). She underwent 6 cycles of monotherapy with methotrexate and achieved remission. The serum HCG level had dropped to negative before the fifth chemotherapy session, and subsequent follow-up HCG tests showed no increase, with standardized follow-up continuing until 2020.

To reduce the risk of recurrent hydatidiform mole, the patient underwent intracytoplasmic sperm injection (ICSI) for assisted reproduction. Both the patient and her husband had normal karyotypes.

The patient was the only child. In her family, there was no consanguinity, no history of similar hydatidiform mole, and no other special genetic disease history. Despite undergoing ICSI for assisted reproduction in 2020, the patient experienced another miscarriage. The product of conception was found to be a triploid.

STR analysis were performed on the products of conceptions including the hydatidiform mole and the ICSI-assisted conception to clarify the parental origin of the product. Meanwhile, the whole-exome sequencing (WES) was also performed for the patient’s family because of the recurrent CHM.

### 2.2. Analysis of microsatellite DNA marker STR polymorphism

Blood samples of the couple and products of conceptions were collected for the analysis of STR polymorphism. Eighteen STR markers, including TH01, D5S818, D7S820, D16S539, CSF1PO, D2S1338, D3S1358, D21S11, D18S51, Penta E, D13S317, Penta D, AMEL, vWA, D8S1179, TPOX, FGA, and D19S433, were used. Markers that do not provide information were excluded. Informative markers including D18S51 and D13S31were used for kinship analysis.

### 2.3. Whole-exome sequencing analysis

Peripheral blood samples from the patient, her husband and her parents were collected, from which genomic DNA was extracted. The entire exome was captured using gene probes, followed by polymerase chain reaction amplification. The resulting amplified products were sequenced on the Illumina high-throughput sequencer. A high average coverage depth (90-110X) was used to cover over 99% of the variants in CCDS, ClinVar, Ensembl (CDS), miRBase, OMIM, RefSeq (CDS), and VEGA (CDS). Variants that were highly or potentially related to the patient’s phenotype were further verified using the Sanger sequencing.

### 2.4. Diagnostic assessment

#### 2.4.1. Pathological analysis

**Figure 1.**
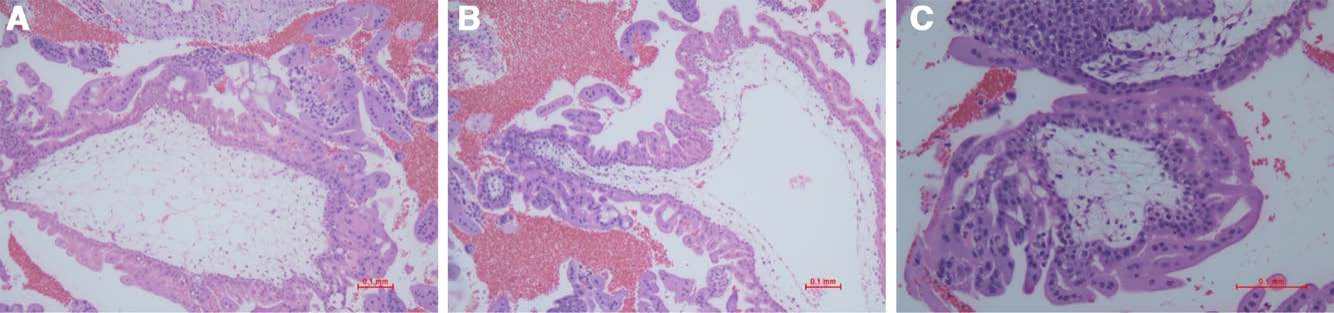
Pathological analysis. (A) hydropic swelling of chorionic villi (low power 100×); (B) hydropic swelling of chorionic villi with central cistern formation, and absence of fetal blood vessels within the villous stroma (low power 100×); (C) trophoblastic hyperplasia and lacking the normal polar arrangement (high power 200×).

In the patient’s first and second pregnancy, the pathological sections of the uterine pregnancy mass exhibited hydropic swelling of chorionic villi with central cistern formation, and absence of fetal blood vessels within the villous stroma. At high power, diffuse trophoblastic hyperplasia was identified. The trophoblastic proliferation was circumferential and disorganized, lacking the normal polar arrangement observed in non-molar villi. These findings were consistent with the diagnosis of CHM (Fig. 1A–C).

#### 2.4.2. Analysis of STR polymorphism

**Figure 2.**
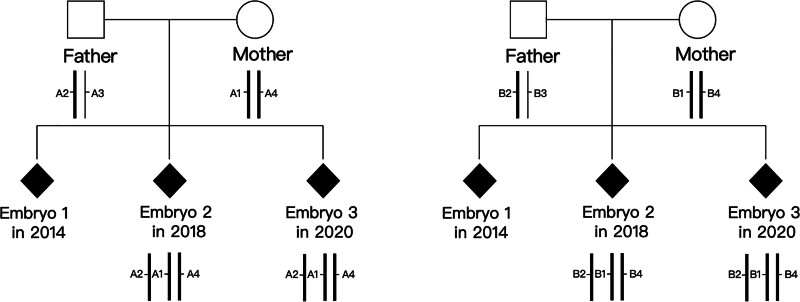
Pedigree chart of short tandem repeat (STR) results. It visually represents the STR analysis results within the family lineage. The chart uses standardized symbols to denote gender (square for male, circle for female, and solid diamond for miscarriage of unknown gender). Group A (left) refers to STR markers D18S51 and group B (right) refers to D13S317. STR = short tandem repeat.

STR polymorphism analysis were conducted on the products of every conception. STR analysis, including markers D18S51, D13S317, D8S1179, and D19S433, for the first hydatidiform mole were not successful. The failure of STR analysis possibly due to the severe fragmentation of deoxyribonucleic acid (DNA) caused by the long-time storage of the paraffin-embedded tissue, which affected the amplification of polymerase chain reaction. In the STR markers D18S51 and D13S317 loci, STR polymorphism analysis for the second hydatidiform mole and the third missed abortion suggested that both of the samples inherited 1 allele from the father and 2 alleles from the mother, a parental origin triploid with double maternal and single paternal alleles (Table 1). Pedigree chart visually represents the STR analysis results in Figure 2.

**Table 1:** Analysis of STR polymorphism for the 3 products of conceptions.

STR markers	STR results					Parental origins
	Father	Mother	First conception in 2014	Second conception in 2018	Third conception in 2020	
D18S51	A2/A3	A1/A4	–	A1/A2/A4	A1/A2/A4	Triploid with double maternal and single paternal alleles
D13S317	B2/B3	B1/B4	–	B1/B3/B4	B1/B3/B4	Triploid with double maternal and single paternal alleles

STR = short tandem repeat.

#### 2.4.3. Analysis of WES

**Figure 3.**
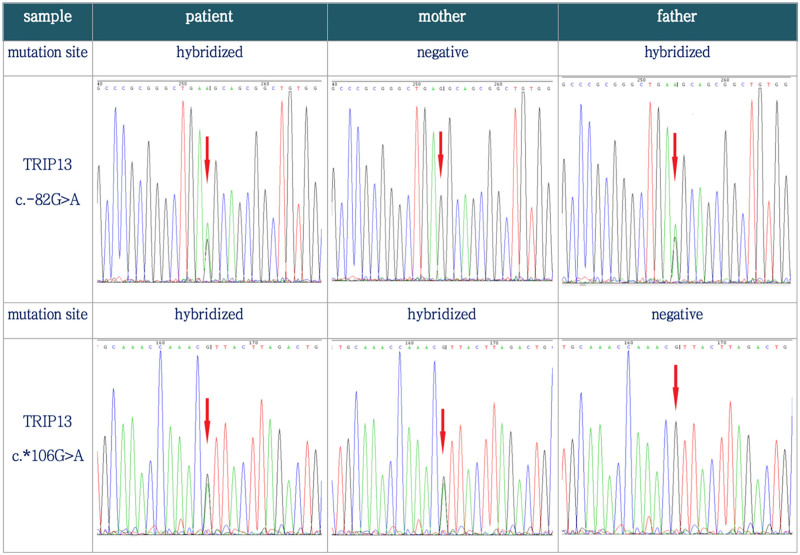
Verification results of Sanger sequencing. The patient had a pair of compound heterozygous variants in the autosomal recessive gene thyroid hormone receptor interactor 13 (*TRIP13*) c.-82G > A and c.*106G > A from her parents. TRIP13 = thyroid hormone receptor interactor 13.

Biparental recurrent hydatidiform mole was reported often linked to autosomal recessive genetic defects. Therefore, we conducted WES on the patient’s family, and the results revealed that the patient has a pair of compound heterozygous variants in the autosomal recessive gene thyroid hormone receptor interactor 13 (*TRIP13*) c.-82G > A (maternally negative, paternally heterozygous) and c.*106G > A (maternally heterozygous, paternally negative; Table 2). Further Sanger sequencing confirmed these findings (Fig. 3). The WES test of her husband was negative.

**Table 2:** Whole exome sequencing results of the patient’s pedigree.

Gene	Inheritance patternt	Mutation coordinates	Mutation description	Results of pedigree
TRIP13	Autosomal recessive	chr5:893032 G > A	NM_004237.4:5UTR c.-82G > A	Maternal negative/paternal hybrid
TRIP13	Autosomal recessive	chr5:917324 G > A	NM_004237.4:3UTR c.*106G > A	Maternal hybrid/paternal negative

TRIP13 = thyroid hormone receptor interactor 13.

#### 2.4.4. Diagnosis

Final diagnosis: *TRIP13* gene mutation; personal history of BiCHM; personal history of adverse pregnancy outcomes.

#### 2.4.5. Therapeutic intervention

In this case, the patient had compound heterozygous mutations in the *TRIP13* gene, experienced 2 molar pregnancies and 1 miscarriage. Specimens were collected successfully, and genetic analysis indicated biparental triploid origins. Considering the patient’s advanced maternal age and compound heterozygous mutation, our clinical recommendations were obtaining offspring through egg donation-assisted reproduction or adoption. Regrettably, the patient abandoned subsequent assisted reproductive treatments including egg donation due to advanced age. To date, she has not had any live births nor pursued adoption.

## 3. Discussion

The patient reported in this case presented with recurrent hydatidiform mole and miscarriage following assisted reproductive technology. Pathological examination indicated that the hydatidiform moles were both CHM. Meanwhile, STR testing revealed that all 3 pregnacy were biparental triploid. Triploid BiCHM represents a clinically rare type of CHM.

According to the existing literature search results, the genes primarily associated with BiCHM were different loci of *NLRP7* or *KHDC3L*, with few reports of other related genes found. *TRIP13* was a widely expressed gene with important functions in meiosis. A recently study in our laboratory found that the *TRIP13*-dependent removal of Helper Of Rec8 Meiosis Activation Domain (HORMAD) 1 and 2 from synapsed chromosome axes was essential for female fertility. When HORMAD1 and HORMAD2 were retained on synapsed chromosome axes, they would activate chromosome asynapsis checkpoint through BRCA1, and trigger oocyte elimination.^[7]^ Previous studies found that defects in the *TRIP13* gene could result in the occurrences of mosaic aneuploidy syndrome type 3 and oocyte maturation defect 9. Mosaic aneuploidy syndrome type 3 is an autosomal recessive disorder caused by chromosome missegregation, with most patients developing early-onset Wilms tumor and cytogenetic aneuploidy abnormalities and premature chromatid separation.^[8]^ Oocyte maturation defect 9 is a disease that causes female infertility due to the arrest of most patients’ oocytes at the metaphase I stage of meiosis or zygote cleavage arrest, and the mode of inheritance is also autosomal recessive. Zhang et al found that injecting *TRIP13* cRNA into the oocytes of an affected individual could rescue the phenotype, but this technology has not yet been approved for clinical treatment.^[9]^

In this case, the patient did not exhibit reported *TRIP13* gene related diseases. However, in meiosis, maternal-derived gametes appeared to experience chromosomal missegregation or nondisjunction, leading to the transmission of maternal diploidy, which might directly result in the formation of a triploid embryo with biparental origin, that is, maternal diploidy and paternal haploidy. Genomic instability and epigenetic alterations may also lead to chromosomal missegregation or nondisjunction. The underlying mechanism require further research. Based on previous research findings, *TRIP13* cRNA injection technology may serve as a promising therapeutic approach in the future.

## Acknowledgments

We are grateful for the participation of the patient, and the technological supports of Human Genetic Variation Group and Medical Genetics Laboratory of Zhejiang University School of Medicine, as well as Yizhen Biotechnology.

## Author contributions

**Writing – review & editing:** Xinyun Yang, Yan Wang.

**Writing – original draft:** Qitao Zhan.
